# Gene expression changes with differentiation of cord blood stem cells to respiratory epithelial cells: a preliminary observation

**DOI:** 10.1186/scrt60

**Published:** 2011-04-13

**Authors:** Michael J Berger, Sharon R Minnerath, Sheryl D Adams, Barbara M Tigges, Stacey L Sprague, David H McKenna Jr

**Affiliations:** 1 Department of Laboratory Medicine and Pathology, University of Minnesota, 420 Delaware Street SE, MMC609, Minneapolis, MN 5545, USA; 2 Fairview Molecular Diagnostics Lab, University of Minnesota, D210 Mayo Building, 420 Delaware Street SE, MMC198, Minneapolis, MN 55455, USA; 3 Molecular Cellular Therapy, University of Minnesota, 1900 Fitch Avenue, St Paul, MN 55108, USA; 4 BioE Inc., 4280 Centerville Road, St Paul, MN 55127, USA

## Abstract

**Introduction:**

Owing to wide availability, low cost and avoidance of ethical concerns, umbilical cord blood (UCB) provides an attractive source of stem cells for investigational and therapeutic uses. In this study, we sought to characterize the gene expression changes as stem cells from UCB differentiate toward alveolar type II pneumocytes (ATII).

**Methods:**

Control and experimental cells were cultured in maintenance medium (mesenchymal stem cell growth medium) or differentiation medium (small airway growth medium (SAGM)), respectively, for 8 days. Total RNA was isolated from control and experimental groups for gene expression profiling and real-time polymerase chain reaction assay.

**Results:**

Analysis of only mixed cell lines (*n *= 2) with parameters including a *P *value of 0.01 and an intergroup gap of 2.0 yielded a set of 373 differentially expressed genes. Prominently upregulated genes included several genes associated with ATII cells and also lung cancers: *ALDH3A1*, *VDR *and *CHKA*. Several upregulated genes have been shown to be integral or related to ATII functioning: *SGK1*, *HSD17B11 *and *LEPR*. Finally, several upregulated genes appear to play a role in lung cancers, including *FDXR *and *GP96*. Downregulated genes appear to be associated with bone, muscle and central nervous system tissues as well as other widespread tissues.

**Conclusions:**

To the best of our knowledge, this accounting of the gene expression changes associated with the differentiation of a human UCB-derived stem cell toward an ATII cell represents the first such effort. Dissecting which components of SAGM affect specific gene regulation events is warranted.

## Introduction

Boyse first proposed the use of human umbilical cord blood (UCB) for therapeutic reconstitution (Boyse EA *et al*., unpublished), and later Broxmeyer *et al. *[1] showed that the frequency of hematopoietic progenitor cells in this graft source surpassed that found in bone marrow. Researchers now commonly utilize UCB as a source of mesenchymal stromal cells (MSCs) [2], and several investigators have reported success in isolating pluripotent stem cells [3,4]. Owing to wide availability, low cost and avoidance of ethical problems, UCB provides an attractive source of stem cells for investigational and therapeutic uses, including cell therapy approaches to treating lung diseases [5,6].

Recently, some of the molecular and cell biological features important in the *in vivo *maintenance of lung stem cells (bronchioalveolar stem cells) and their differentiation into lung epithelium have been described [7]. Wade *et al. *[8] documented the gene expression changes engendered by treatment of cultured fetal lung epithelial cells with dexamethasone and cyclic AMP (cAMP) during differentiation into alveolar type II pneumocytes (ATII) and identified a set of "hormonally responsive" genes putatively involved in this process. Previously we reported the differentiation of UCB-derived multilineage progenitor cells (MLPCs) into cells which expressed surfactant protein C (SPC) mRNA as well as SPC and which displayed morphologic features (visualized by light and transmission electron microscopy) consistent with ATII cells [9]. However, in general, the precise mechanisms underpinning *in vitro *differentiation events involving stem cells remain obscure.

A more complete understanding of these differentiation processes may aid in the development of cell-based therapeutic approaches and enhance our knowledge of progenitor cell biology. In the current study, we report the results of gene expression profiling performed on our previously described, UCB-derived multipotent stem cells, or MLPCs, differentiated in culture into cells that express features of ATII cells [9].

## Materials and methods

### Cell culture

MLPCs were cultured and differentiated in small airway growth medium (SAGM; Lonza, Walkersville, MD, USA) as described earlier [9]. RNA was extracted from three pairs of control (cells maintained in standard stem cell medium) and SAGM-cultured cells using TriReagent (Molecular Research Center, Inc., Cincinnati, OH, USA), representing two mixed and one clonal MLPC lines.

### Microarray analysis

Following the Affymetrix GeneChip Human Genome U133A Plus 2.0 protocol (Affymetrix, Santa Clara, CA, USA), total RNA was converted to biotin-labeled cRNA, which was then hybridized to U133A Plus 2.0 Affymetrix microarray chips by the technical staff at the University of Minnesota Microarray Facility, a part of the BioMedical Genomics Center [10]. All washing, staining and scanning procedures were performed as described in the Affymetrix protocols [11]. The U133A Plus 2.0 contained 47,000 probe sites, with multiple redundancies at selected loci. A total of six hybridization events were performed, beginning with RNA from three differentiated lines and three control cell lines.

The raw fluorescence data (in Affymetrix CEL file format) containing fluorescence readings for 11 pairs of 24- to 30-bp probes with one perfect match and one mismatched probe in each set were analyzed using Expressionist software downloaded from the Minnesota Supercomputing Institute website at the University of Minnesota [12]. The data were normalized for overall expression level to a median reference value of 10,000 dimensionless fluorescence units. The data from all hybridizations had the same median fluorescence values before normalization, and this step was unnecessary. Differentially regulated genes were identified using the *t*-test within the Expressionist software analysis package. The microarray data were deposited into ArrayExpress (accession number E-MEXP-3041) [13].

### Statistical analysis

Genes were selected as up- or downregulated using the *t*-test as described above with *P *values less than or equal to 0.01 and an intergroup gap greater than or equal to 2.0. Additional analyses of the entire expression set were performed with *P *values ranging from 0.01 to 0.0001.

### Database analysis

Details regarding the disease associations for each gene of interest were gathered from the Online Mendelian Inheritance in Man database [14]. Assignment of molecular function was derived from the Swiss-Prot database [15].

### Real-time reverse transcriptase polymerase chain reaction assay

Total RNA was isolated and cDNA was synthesized as described earlier [9]. Primers and probes for each gene analyzed were obtained from Applied Biosystems (Foster City, CA, USA). Protocols utilized standard manufacturer-recommended conditions for the TaqMan Gene Expression Assay (Life Technologies, Foster City, CA, USA) chemistry employed. Glyceraldehyde 3-phosphate dehydrogenase (*GAPDH*) was amplified as a housekeeping control. The amount of target gene expression was normalized to the expression of *GAPDH *and to a calibrator using the comparative cycle threshold (*C*_t_) method, 2^-ΔΔ*C*^_t_, as described in detail in User Bulletin 2 for the ABI Prism 7700 Sequence Detection System (Applied Biosystems).

## Results

### General gene expression profiling results

Analysis of the raw data generated from the gene expression chip hybridization showed 23,000 analyzable data points. At *P *values ranging from 0.01 to 0.0001, the number of differentially expressed genes between the induced and control groups exceeded that expected by chance by approximately three- to sixfold (see Table 1).

**Table 1:** Differentially expressed genes at various *P *values^a^

*P *value	Differentially expressed genes	Expected
0.01	845	230
0.001	71	23
0.000	12	2

^a^Using Expressionist software and analyzing the mixed cell lines, the numbers of differentially expressed genes are listed for various *P *values.

Analysis of only mixed lines with parameters including a *P *value of 0.01 and an intergroup gap of 2.0 yielded a set of 373 differentially expressed genes. Analysis of the clonal cell line yielded a similar set of genes. After exclusion of expressed sequence tags and unknown genes, we characterized the function of 163 induced and 108 downregulated genes. The induced genes represented a wide range of molecular functions, including transcription regulation, DNA binding, transport, RNA binding, signaling and housekeeping (see Table 2). Similarly, the downregulated genes comprised a diverse functional group and included transcription factors, DNA binding, transport, RNA binding, signaling and housekeeping (see Table 3). Polycomb group (PcG)-associated genes accounted for 8 (4.9%) of 162 induced genes, including the three most upregulated genes, and 12 (11.1%) of 108 downregulated genes.

**Table 2:** Molecular functions of upregulated genes^a^

Molecular function	MLPCs (induced)	Genome
Transcription regulation	15.4	10.4
Hydrolase	10.4	8.7
Unknown function	10.4	
RNA binding	9.9	
Protein binding	9.2	
DNA binding	6.2	9.1
Transport	4.9	5.9
Signaling	4.9	21.1
Oxidoreductase	4.3	3.8
Receptor	4.3	4.7
Kinase	3.7	1.1
Transferase	3.1	8.2
Chaperone	2.5	
Ion transport	2.5	1.9
Ligase	1.9	
Developmental	1.2	2.8
Membrane protein	1.2	
Protein synthesis	1.2	
Metal binding	0.6	6.2
Nuclear anchor	0.6	
Immune modulation	0.6	
Protease inhibitor	0.6	

^a^MLPC, multilineage progenitor cell. Molecular functions of genes upregulated in small airway growth medium-induced cells, as defined in the Swiss-Prot and TrEMBL databases, expressed as a percentage of the total upregulated group and compared to molecular functions of all known genes in the genome (see Wade *et al. *[8]).

**Table 3:** Molecular functions of downregulated genes as defined in the Swiss-Prot and TrEMBL databases, expressed as a percentage of the total downregulated group and compared to molecular functions of all known genes in the genome^a^

Molecular function	MLPCs (induced)	Genome
Transcription regulator	6.5	10.4
Developmental	6.5	2.8
Receptor	6.5	4.7
Signaling	4.6	21.1
Hydrolase	4.6	8.7
Cell adhesion	4.6	0.4
Transport	4.6	5.9
Muscle differentiation-related	4.6	
Unknown function	4.6	
Extracellular matrix	3.7	1.4
RNA binding	3.7	
Protein binding	3.7	
Cytoskeletal	3.7	
Oxidoreductase	2.8	3.8
GTPase	2.8	10.6
Transferase	2.8	8.2
Calcium binding	2.8	3.1
Isomerase	1.9	0.7
Chromatin regulator	1.9	
Binding	1.9	
Ligase	1.9	
Cell junction	1.9	
Metal binding	1.9	6.2
Kinase	1.9	1.1
Growth factor	0.9	
Secreted	0.9	
Calmodulin-binding	0.9	1
G protein exchange	0.9	
Membrane protein	0.9	
Cytokine	0.9	
Motor protein	0.9	
ATP binding	0.9	7.3
Hormone	0.9	
Tumor antigen	0.9	
RNA splicing	0.9	
Endosomal trafficking	0.9	
ATP synthesis	0.9	
Ion channel	0.9	0.2
Lysase	0.9	0.9

^a^MLPC, multilineage progenitor cell. Molecular functions for which no genome value is listed reflect updates in the Swiss-Prot/TrEMBL databases and author discretion in assigning function if no clear function is listed in the above-mentioned databases (see Wade *et al. *[8]).

Tables 4 and 5 show the most prominently up- and downregulated, as well as selected, genes of interest. Table 6 details gene chip and quantitative real-time reverse transcriptase polymerase chain reaction (qRT-PCR) assay data for a subset of hormonally responsive genes described by Wade *et al. *[8] (see Introduction).

**Table 4:** Upregulated genes^a^

Official symbol	Name	Fold change	Significance
*ALDH3A1*	Aldehyde dehydrogenase 3 family member A1	13.2	Expressed in rat lung and non-small cell lung cancers. May contribute to cyclophosphamide resistance.
*FDXR*	Ferredoxin reductase	9.8	Higher expression levels seen in metastatic colorectal cancer than in normal controls.
*GADD45B*	Growth arrest and DNA damage-inducible β	7.6	Induced by differentiation-inducing cytokines.
*SGK1*	Serum/glucocorticoid-regulated kinase 1	5.0	Expressed in lung, functioning in water and sodium balance. Mineralocorticoid-responsive.
*VDR*	Vitamin D (1,25-dihydroxyvitamin D_3_) receptor	4.6	Hypothesized to play a key role in ATII maturation and surfactant synthesis.
*HSP90B1*	Heat shock protein 90 kDa β (Grp94)	4.2	Increased expression seen in mesothelioma and squamous cell lung cancer.
*HDS17B1*	Hydroxysteroid (17β) dehydrogenase 11	3.6	Expressed in distal bronchioles and ATII cells.
*CHKA*	Choline kinase α	3.4	Expressed in adult rat ATII cells. Forms part of synthesis pathway for phosphatidylcholine (pulmonary surfactant synthesis).

^a^ATII, alveolar type II pneumocyte. Expression change (expressed as fold change relative to control cells) and significance of selected upregulated genes.

**Table 5:** Downregulated genes^a^

Official symbol	Name	Fold change	Significance
*CTHRC1*	Collagen triple-helix repeat containing 1	37.5	Inhibitor of TGF-β
*INHBA*	Inhibin, βA	26.4	Part of TGF-β superfamily with mitogenic effects on osteoblasts
*GRB14*	Growth factor receptor-bound protein 14	23.8	Negative regulator of insulin receptor signaling
*SCRG1*	Stimulator of chondrogenesis 1	15.6	Cartilage formation
*PDE1C*	Phosphodiesterase 1C, calmodulin-dependent	10.8	Phosphodiesterase highly expressed in heart and brain
*SRY-BOX 11*	SRY (sex-determining region Y) box 11	9.1	Possible role in developing nervous system
*COL4A*	Collagen type IV, α_1_	8.3	Basement membrane collagen
*DOCK 10*	Dedicator of cytokinesis 10	7.8	G protein exchange factor with widespread expression
*PDLIM7*	PDZ and LIM domain 7	7.8	Mediator of protein exchange with ubiquitous expression
*CYP4V2*	Cytochrome P450, family 4, subfamily V polypeptide 2	7.3	Mutated in Bietti crystalline dystrophy
*CD44*	CD44 molecule	2.2	Crucial for stem cell niche interactions

^a^TGF, transforming growth factor. Expression change (expressed as fold change relative to control cells) and significance of selected downregulated genes.

**Table 6:** Hormone-responsive and additional genes of interest: GeneChip and real-time PCR data^a^

Official symbol	Name	qRT-PCR I/C	GeneChip I/C	Significance
*SBP*	Surfactant protein B	NA	-0.6	Putative HR gene
*LAMP3*	Lysosomal associated membrane protein 3	0.03	1.0	HR
*LPL*	Lipoprotein lipase	1.43	25.0	HR
*SERPINA1*	α_1 _antiproteinase, antitrypsin	1.7	15.0	Secreted by ATII cells; functional importance
*ABCA3*	ATP-binding cassette, subfamily A, member 3	7.9	100.0	Expressed in lung; functions in surfactant production
*HIF3a*	Hypoxia-inducible factor 3, α-subunit	7.9	60.0	HR
*MAOA*	Monoamine oxidase A	10.0	25.0	HR
*ABCA1*	ATP-binding cassette, subfamily A, member 1	103.2	6.0	Expressed in lung; functions in surfactant production
*NR4A2*	Nuclear receptor subfamily 4, group A, member 2	110.7	5.0	HR
*ALDH1b*	Aldehyde dehydrogenase 1β	10,716.0	2.0	HR
*GAPDH*	Glyceraldehyde 3-phosphate dehydrogenase	1.1	NA	Housekeeping gene

^a^PCR, polymerase chain reaction; qRT-PCR, quantitative real-time polymerase chain reaction; I/C, ratio of induced to control cells; HR, hormone-responsive; ATII, alveolar type II pneumocyte; NA, not applicable. GeneChip and quantitative PCR data are expressed as ratios of induced to control cells and the context (that is, significance) of this gene.

## Discussion

To the best of our knowledge, this account of the gene expression changes associated with the differentiation of a human UCB-derived stem cell toward a terminally differentiated respiratory epithelial cell (namely, an ATII cell) represents the first such effort. Our control group consisted of multipotential human cord blood-derived stem cells (MLPCs) maintained in a standard mesenchymal stem cell growth medium (MSCGM; Stem Cell Technologies, Vancouver, BC, Canada). Our experimental group utilized the same cells cultured in an airway epithelium maintenance medium (SAGM; Stem Cell Technologies). Few studies exist for comparison, and these groups studied expression changes from different starting cell populations. For example, Wade *et al. *[8] isolated human second trimester lung epithelial cells and differentiated these cells in culture using various combinations of cAMP and dexamethasone as differentiation agents. Despite a recent report [16] detailing surface immunophenotype similarities between neonatal lung cells and mesenchymal stem cells, we anticipated major differences in the gene expression profiles between our cells: (1) human UCB-derived cells differentiated toward ATII cells and (2) human lung cells.

Using Expressionist software to analyze the raw hybridization data, three to six times the number of genes appear to be reproducibly differentially expressed relative to what would be expected by chance. This finding suggests that a differentiation event has taken place and that we are not simply viewing "noise" in the system. Similarly, the percentages of the upregulated and downregulated genes in each molecular function category generally mirror the percentages of these categories among known genes in the entire genome, with several notable departures. In both the up- and downregulated sets, the percentages of genes that effect signaling and metal binding are lower than those in the genome as a whole. In the downregulated gene group, the percentage of genes that function in cell adhesion is higher than that found among all genes. These data suggest that signaling and metal binding functions are similar for MLPCs and the differentiated counterpart, and therefore are likely equivalently crucial for maintenance of both phenotypes. The decrease in the expression of cell adhesion genes in the induced cells may indicate a change in the repertoire of possible extracellular matrix interactions between the induced and control cells.

### Stem cell differentiation and regulation

Epigenetic modifications, such as methylation of lysine residues on histone proteins (H3K27me3 and H3K4me3), play a major role in differentiation and cell fate determination and are effected via the actions of the PcG proteins [17]. Recent reports indicate that genes that feature only the H3K4 mark (allowing gene expression) show high expression and function primarily in the maintenance of metabolism. A second group of genes features both the H3K4 mark (allowing expression) and the H3K27 mark (contributing to a repressive chromatin state), thus creating a "bivalent state" poised for either down- or upregulation. This latter group of genes functions as regulators of development. A small percentage of genes contain only the H3K27 mark, and the substantial balance of genes contain neither; these latter genes are thought to be upregulated in response to physiologic stimuli [18]. Comparison of our set of regulated genes with *SUZ12*-associated genes (*SUZ12 *participates in the complex responsible for creating the H3K27me3 mark; the bulk of the genes marked in this way exist in the "bivalent state") [18,19] shows a greater number of *SUZ12*-associated genes in the downregulated relative to the upregulated set (11% versus 4.9%). This result meets expectation that as stem cells differentiate, an increasingly select group of genes is expressed. Further, the top three upregulated genes in our set are part of the *SUZ12*-associated set, suggesting the possibility that these genes are developmentally important genes that may have held dual (activating and repressing) epigenetic marks and were upregulated in response to the differentiating agents. It should be noted that the lists of PcG-associated genes were generated via analyses of human and mouse embryonic stem cells and that such data for human cord blood stem cells have not been thus far reported [18,20,21].

### Upregulated genes

The upregulated gene set includes three genes with documented expression and function in ATII: *SGK1*, *HSD17B11 *and *LEPR*. Webster *et al. *[22] found high expression of *SGK1 *in the lung as well as in other tissues, and other researchers [23] have demonstrated induction of *SGK1 *via the actions of mineralocorticoid receptors. ATII cells function in the lung as the primary site for reabsorption of excess sodium, and *SGK1 *may function as a regulator of this process, possibly through phosphorylation of inducible nitric oxide synthase [24,25]. *HSD17B11*, an enzyme possibly involved in androgen metabolism, displays expression in fetal and adult distal bronchioles as well as in ATII cells [26].

In a separate analysis of induced versus control cells, which involved both mixed and clonal MLPC lines, the induced cells expressed approximately 10 times the mRNA for *LEPR *relative to controls. Leptin, produced by adipocytes, is thought to regulate body fat via a neural feedback mechanism. Recent work by Bergen and colleagues [27] demonstrates the presence of functional leptin receptors in both adult and fetal rabbit lung and further refines the location of expression to include acinar epithelial cells and ATII cells. Their study also showed that increasing concentrations of leptin resulted in increased production of disaturated phosphatidylcholine- a specific marker of pulmonary surfactant production.

A second set of upregulated genes includes three genes that function in ATII cells and have also been associated with lung cancers: *ALDH3A1*, *VDR *and *CHKA*. Aldehyde dehydrogenases function in the lung to break down aldehydes which may occur naturally in the environment as a breakdown product of xenobiotics or as part of endogenous metabolism. Yoon *et al. *[28] found that the expression of the specific isoform *ALDH3A1 *was restricted to the lung (relative to the liver) and that the level of expression of this gene increased from day 1 to day 60 in Wistar Han rats. Other recent work in humans demonstrated *ALDH3A1 *and *ALDH1A1 *expression by immunohistochemistry in squamous cell carcinomas (SSCAs) and adenocarcinomas (AdenoCAs), but not in small cell lung cancer (SCLC) [29]. This same group showed increased expression of these genes in the pneumocytes of smokers relative to nonsmokers; both proteins were expressed at high levels in normal bronchial epithelium as well [29]. The fact that SSCAs and AdenoCAs express *ALDH3A1 *and *ALDH1A1 *takes on importance in the treatment phase, as the presence of these enzymes has been associated with resistance to oxazaphosphorines such as cyclophosphamide. Muzio *et al. *[30] and Moreb *et al. *[31] have shown in A549 cell culture models that introducing arachidonic acid or selectively knocking down RNA expression of ALDH3A1 and/or ADLH1A1 causes increased susceptibility to cyclophosphamide.

Vitamin D receptors (*VDR*) appear around days 19 to 21 in studies of fetal rat lung [32]. Investigations of rat lungs using electron immunogold labeling show that *VDR *appears to be restricted to ATII cells. Mediated via the downstream effects of engaged *VDR*, 1,25(OH)_2_D_3 _(calcitriol) plays a key role in ATII maturation and pulmonary surfactant synthesis, the latter possibly by modulation of fructose 1,6-bisphosphatase mRNA expression [33]. Currently, the antiproliferative and chemopreventative properties of vitamin D and metabolites are being evaluated in the setting of various neoplasms, including lung cancer. Calcitriol has been shown to slow lung cancer tumor growth and metastases in mouse models [34].

Phosphatidylcholine forms the basis for pulmonary surfactant: adult rat ATII cells show expression of *CHKA*, coding for an enzyme in the synthesis pathway for this molecule [35]. *CHKA *overexpression may play a role in cell proliferation and carcinogenesis, and recent work suggests that high expression of this protein may negatively impact patient prognosis in human lung cancers [36].

A third set of upregulated genes includes two genes that have been associated with various cancers: *FDXR *and *HSP90B1. FDXR *functions in the p53-dependent apoptosis pathway. Ichikawa *et al. *[37] showed that in metastatic colorectal cancer, expression of *FDXR *was higher in tumors that responded to therapy with 5-fluorouracil than in nonresponders. Adjuvant compounds that cause increased expression of *FDXR *may be useful in this setting. Mesothelioma and squamous cell lung cancer cells express higher levels of *HSP90B1 *mRNA than matched normal controls [38,39].

Additional upregulated genes do not appear to be related to either ATII development or function or lung cancers. For example, *GADD45B *binds to nuclear hormone receptors (such as the retinoic acid receptor) and is induced by various stressors, including differentiation-inducing cytokines [40]. The role of *GADD45B *expression in differentiation is currently under investigation.

In summary, the most prominently upregulated genes include three genes (*SGK1*, *HSD17B11 *and *LEPR*), which have been found in previous studies to be expressed in ATII cells and likely contribute to the functioning of this cell type. Three genes (*ALDH3A1*, *VDR *and *CHKA*) are associated both with ATII physiology and lung cancers. Two genes (*FDXR *and *HSP90B1*) are associated with cancer: *FDXR *with colorectal cancer and *HSP90B1 *with SCLC and mesothelioma. These data corroborate our earlier findings characterizing the differentiation of MLPCs to ATII-like cells [9]. Further, the upregulation of genes that appear to be associated both with normal lung development and/or biology (ATII functioning) and with lung or other cancers is consistent with growing evidence that tissue-specific stem cells appear to underlie some lung cancers [41]. Evaluating the role of the ingredients within SAGM in the perturbations in gene expression may be helpful in advancing our understanding of both normal lung development (stem cell to ATII differentiation) and lung oncogenesis.

### Downregulated genes

The substantially downregulated set of genes includes genes with a mixture of putative functions. Specifically, genes active in bone formation, muscle cell activity and central nervous system development and/or metabolism are represented (for details, see Table 5). Of note, *CD44 *is downregulated in SAGM-induced cells relative to controls. *CD44 *plays key roles in stem cell stromal interactions in general. Wagner *et al. *[42] characterized CD44-stromal cell interactions in CD34^+^/CD38^- ^progenitor cells, particularly in those culled from UCB. Understanding progenitor-stromal cell interactions gains importance when one considers that these interactions are crucial for maintenance of "stemness" properties such as self-renewal and asymmetrical cell division. The leukemic stem cell likewise requires such niche interactions, and this requirement formed the basis for the hypothesis that altering these interactions may form the basis for antitumor therapies. Jin *et al. *[43] showed that the use of an anti-CD44 antibody blocked engraftment of human acute myelogenous leukemia cells transplanted into nonobese diabetic-severe combined immunodeficiency mice. Further, they demonstrated drastically reduced serial repopulation.

### qRT-PCR hormonally responsive gene set

*LPL*, *HIF3a*, *MAOA*, *NR4A2 *and *ALDH1b *showed increased expression (as determined via qRT-PCR assay) in the induced versus control MLPCs (see Table 6). *LAMP3 *showed decreased expression in the induced versus control cells. Wade *et al. *[8] described a set of genes expressed in ATII precursor cells culled from human fetal lungs treated with various combinations of dexamethasone and cAMP. We show upregulation of a subset of these genes in our system, possibly indicating a basic role for these genes in the stem cell to ATII cell transition.

### qRT-PCR for additional genes of interest

*SERPINA1 *(α_1_-antitrypsin), *ABCA1 *and *ABCA3 *showed increased expression in our induced versus control cells by qRT-PCR assay (see Table 6). α_1_-Antitrypsin is secreted by ATII cells, functions to maintain protease balance and further plays a key role in the pathophysiology of emphysema. Understanding the regulation of this gene may provide insights into emphysema treatment. *ABCA1 *and *ABCA3 *code for ATP-binding cassette proteins that transport lipids and sterols and are integral to surfactant production. These proteins are found in the limiting membrane of the lamellar bodies, and, further, *ABCA3 *mRNA is highly expressed in the lung. The upregulation of these genes is consistent with our hypothesis that induced MLPCs have adopted features of ATII cells and further adds to our understanding of the stem cell-to-ATII cell transition.

Finally, our data may provide some of the tools used to improve the differentiation of UCB-derived stem cells to ATII cells. Samadikuchaksaraei and Bishop [44], using murine embryonic stem cells, determined that a serum-free medium, small airway basal medium (SABM; SAGM minus all additives), stimulated the production of SPC mRNA more robustly than SABM with single additions of any of the numerous additives that combine to make up SAGM. Building on this work, we think that it may be instructive to utilize additional and alternative end points to SPC mRNA expression as a means of monitoring the effectiveness and completeness of a differentiating agent such as SAGM. Our study suggests numerous possible genes or combinations of genes the monitoring of which may provide subtle information regarding cell state.

ATII cells play multiple roles in distal lung processes. Besides the formation of pulmonary surfactant and the regulation of fluid and ionic balance, ATII cells can proliferate in response to lung injury and differentiate into alveolar type I pneumocytes to replace injured epithelium. Ultimately, UCB-derived stem cells, differentiated *in vitro *into ATII cells, may form the basis of a therapeutic approach to repair diseased or injured lung tissue. Understanding the genetic mechanisms underpinning UCB stem cell to ATII differentiation may play a crucial role in the success of this effort.

## Conclusions

In summary, the gene expression data described in this article show parallels with earlier efforts and substantially extend our knowledge of the gene expression changes that accompany the differentiation of MSC-like UCB cells with a hormone cocktail. We anticipate further refinement of these processes, such as may be gathered via culture of our MLPCs in selected elements found within SAGM, serial time points and various alternative culture strategies (for example, Matrigel (BD Biosciences, Franklin Lakes, NJ, USA) and air fluid interface).

## Abbreviations

ATII: alveolar type II pneumocyte; MLPC: multilineage progenitor cell; MSC: mesenchymal stromal cell; SAGM: small airway growth medium; UCB: umbilical cord blood.

## Competing interests

MJB and DHM received research funding for this study from BioE Inc, St Paul, MN, USA. SLS and BT received salaries from BioE Inc, during the time of this study. A patent is pending on the differentiation of MLPCs (cord blood stem cells) to ATII with SAGM (MJB, DHM and BT).

## Authors' contributions

MJB designed experiments, acquired and analyzed data and drafted the manuscript. SRM designed experiments, acquired and analyzed data and reviewed the manuscript. SA designed experiments and reviewed and revised the manuscript. BMT conceived experimental designs and revised the manuscript. SLS conceived experimental designs and revised the manuscript. DHM designed experiments, acquired and analyzed data and drafted and revised the manuscript. All authors read and approved the final manuscript.
